# 可吸收再生氧化纤维素在肺癌手术中的临床效果评价

**DOI:** 10.3779/j.issn.1009-3419.2020.101.10

**Published:** 2020-06-20

**Authors:** 文峰 俞, 金明 徐, 宏旭 盛, 金林 曹, 志田 王, 望 吕, 坚 胡

**Affiliations:** 310003 杭州，浙江大学医学院附属第一医院胸外科 Department of Thoracic Surgery, the First Affiliated Hospital of Zhejiang University, Zhejiang University School of Medicine, Hangzhou 310003, China

**Keywords:** 可吸收再生氧化纤维素, 止血材料, 胸腔镜, 肺肿瘤, Absorbable regenerated oxidized cellulose, Hemostatic material, Thoracoscopic, Lung neoplasms

## Abstract

**背景与目的:**

胸腔镜下安全有效的止血是开展胸外科快速康复的重要条件，术中放置止血材料是肺癌腔镜手术中常用的方法，其中可吸收再生氧化纤维素是常用的止血材料。本研究旨在观察可吸收再生氧化纤维素在肺癌手术中的止血效果。

**方法:**

回顾性分析2018年7月1日-2018年12月1日于浙江大学医学院附属第一医院胸外科行胸腔镜肺癌根治手术且术中使用可吸收再生氧化纤维素止血的42例患者的临床病理资料，选取围手术期指标作为结局事件进行统计分析。

**结果:**

平均手术时间为（120.5±57.3）min，术中平均出血量为（26.8±21.6）mL，术后平均引流量为（513.6±359.5）mL，术后胸管平均留置时间为（2.6±1.2）d。

**结论:**

胸腔镜肺癌根治术术中使用可吸收再生氧化纤维素具有良好的止血效果，适用于淋巴结清扫后创面填塞止血。

## Abstract (en)

**Background and objective:**

Thoracoscopic safe and effective hemostasis is an important condition for rapid rehabilitation of thoracic surgery. Placing hemostatic materials during surgery is a commonly used method in lung cancer laparoscopic surgery. Among them, resorbable oxidized cellulose is a commonly used hemostatic material. This research aims to observe the hemostatic effect of resorbable oxidized cellulose in lung cancer surgery.

**Methods:**

A retrospective analysis of 42 patients with thoracoscopic lung cancer undergoing radical surgery in the Department of Thoracic Surgery, First Affiliated Hospital of Zhejiang University School of Medicine from July 1, 2018 to December 1, 2018, and intraoperative use of regenerative oxidized cellulose to stop bleeding The clinical and pathological data were selected and the perioperative indicators were selected as the outcome events for statistical analysis.

**Results:**

The mean operative time was (120.5±57.3) min. The mean intraoperative blood loss was (26.8±21.6) mL. The average postoperative drainage volume was (513.6±359.5) mL. The average postoperative chest tube indwelling time was (2.6±1.2) d.

**Conclusion:**

The use of absorbable regenerated oxidized cellulose in the radical operation of thoracoscopic lung cancer has a good hemostasis effect, and is suitable for hemostasis of wounds after lymph node dissection.

肺癌在全世界和中国的发病率和死亡率已经高居首位^[1, 2]^，目前手术切除仍是早期肺癌的一线治疗手段。自20世纪90年代胸腔镜（video-assisted thoracic surgery, VATS）手术应用于胸外科领域以来，现今VATS辅助的肺癌根治术已经成为早期肺癌的首选^[3, 4]^，经过近20年的发展，VATS技术已经成为了一项成熟的技术，在此基础上又开展了机器人辅助下胸腔镜技术等。胸腔镜手术相对于传统开放手术更符合现行胸外科加速康复外科的理念，有利于推进胸外科无管化进程^[5]^。现代器械、材料、设备的进展保证了胸外科胸腔镜手术的飞速发展，在微创技术的基础上，加速康复外科的理念已经在胸外科得到广泛实践，并且取得了巨大成就。胸腔镜下有效安全地止血、减少术中出血及减少术后引流量，是开展胸外科快速康复的必要条件^[6]^。据报道，在肺癌术中清扫淋巴结的过程中应用可吸收再生氧化纤维素可以覆盖不规则的创面和潜在出血点^[7]^。本文旨在研究本中心可吸收再生氧化纤维素在肺癌手术中的临床效果，评价其对围手术期指标的影响，为进一步探索止血材料在胸外科快速康复中的作用奠定基础。

## 1 资料与方法

### 1.1 临床资料

连续回顾性纳入2018年7月1日-2018年12月1日浙江大学医学院附属第一医院胸外科行胸腔镜辅助下肺癌根治术且术中使用可吸收再生氧化纤维素的42例患者的临床资料，肺癌的诊断及分期依据美国癌症联合会（American Joint Committee on Cancer, AJCC）肿瘤原发灶-淋巴结-转移（tumor-node-metastasis, TNM）第八版分期标准。手术模式为：VATS或机器人辅助肺癌根治术。手术方式为：肺叶/亚肺叶切除+纵膈淋巴结清扫/采样。患者年龄大于18岁且小于85岁。

本研究中，可吸收氧化再生纤维素为强生公司生产的雪花速即纱。入组患者基本临床特征见表 1。

**1:** 回顾性分析患者的病例资料（*n*=42） Clinical characteristics of patients (*n*=42)

Category	Data
Age (yr)	58.7±10.6
Gender	
Male	10 (23.8%)
Female	32 (76.2%)
Height (cm)	161.0±6.8
Weight (kg)	60.5±10.5
Smoking	2 (4.8%)
Drink	0 (0%)
Hypertension	7 (16.7%)
Diabetes	0 (0%)
Heart disease	0 (0%)
Surgical method approach	
Lobectomy	23 (54.8%)
Sublobectomy	19 (45.2%)
Tumor size (cm)	1.6±1.0

### 1.2 手术方法

患者采用全身麻醉下气管插管，单肺通气，取左侧卧位或右侧卧位，常规消毒铺巾，根据患者自身情况选择单孔（第4肋）或三孔（第7、第4、第9肋间）腔镜下手术，选择肺楔形切除或者肺叶切除术，并行系统淋巴结清扫或系统淋巴结采样，部分多发肺结节患者采取联合肺楔切或联合肺段切除术，保证切缘。止血过程中，将可吸收再生氧化纤维素置于创面渗血处、淋巴结清扫处或切缘处。术后常规使用抗感染、雾化、营养支持等对症支持治疗。

### 1.3 观察指标

记录术后的24 h、48 h、72 h引流量及总引流量、胸管拔除时间以及术后第1天的疼痛评分（参考NRS标准）。

### 1.4 统计学分析

采用SPSS 19.0进行统计学分析，计数资料采用率（%）表示，组间比较采用*χ*^2^检验；计量资料采用均数±标准差（Mean±SD）表示，组间比较采用*t*检验，以*P* < 0.05为差异有统计学意义。

## 2 结果

### 2.1 肺癌患者的围手术期指标

在使用胸腔镜辅助下行肺癌根治术的患者中，患者术后第一天引流量为（212.4±105.3）mL，第二天引流量为（210.1±116.9）mL，第三天引流量为（143.4±96.1）mL，术后总引流量为（513.6±359.5）mL，患者术后拔管时间为（2.6±1.2）d；疼痛评分为（1.4±0.53）分。所有患者均未出现严重的围术期并发症。见表 2。

**2:** 肺癌患者的围手术期指标（Mean±SD） Perioperative indicators for lung cancer patients (Mean±SD)

Category	Data
First day diversion (mL)	212.4±105.3
Second day diversion (mL)	210.1±116.9
Third day diversion (mL)	143.4±96.1
All diversion (mL)	513.6±359.5
Drainage (d)	2.6±1.2
Pain score	1.4±0.5
Lymph node	7.9±4.7
Surgery duration (min)	120.5±57.3
Hemorrhage (mL)	26.8 ±21.6

### 2.2 典型病例展示

《胸外科围手术期出血防治专家共识》中提及，当淋巴结清扫创面不规则时，能量器械又难以施展时，可使用再生氧化纤维素等止血材料填塞。66岁男性，因体检发现右肺结节4年余入院，无慢性病史，无吸烟、饮酒史，无恶性肿瘤病史，排除手术禁忌后行VATS右肺上叶切除+右肺下叶楔形切除+纵隔淋巴结清扫+肺大疱切除修补术，术中清扫第2、4组淋巴结，创面渗血，此处位置使用单纯电凝止血需跨过奇静脉弓进行操作，具有一定难度和风险，术中使用可吸收再生氧化纤维填塞既可达到止血的目的，又避免大面积电凝止血对附近组织的损伤风险，如图 1所示。

**1:**
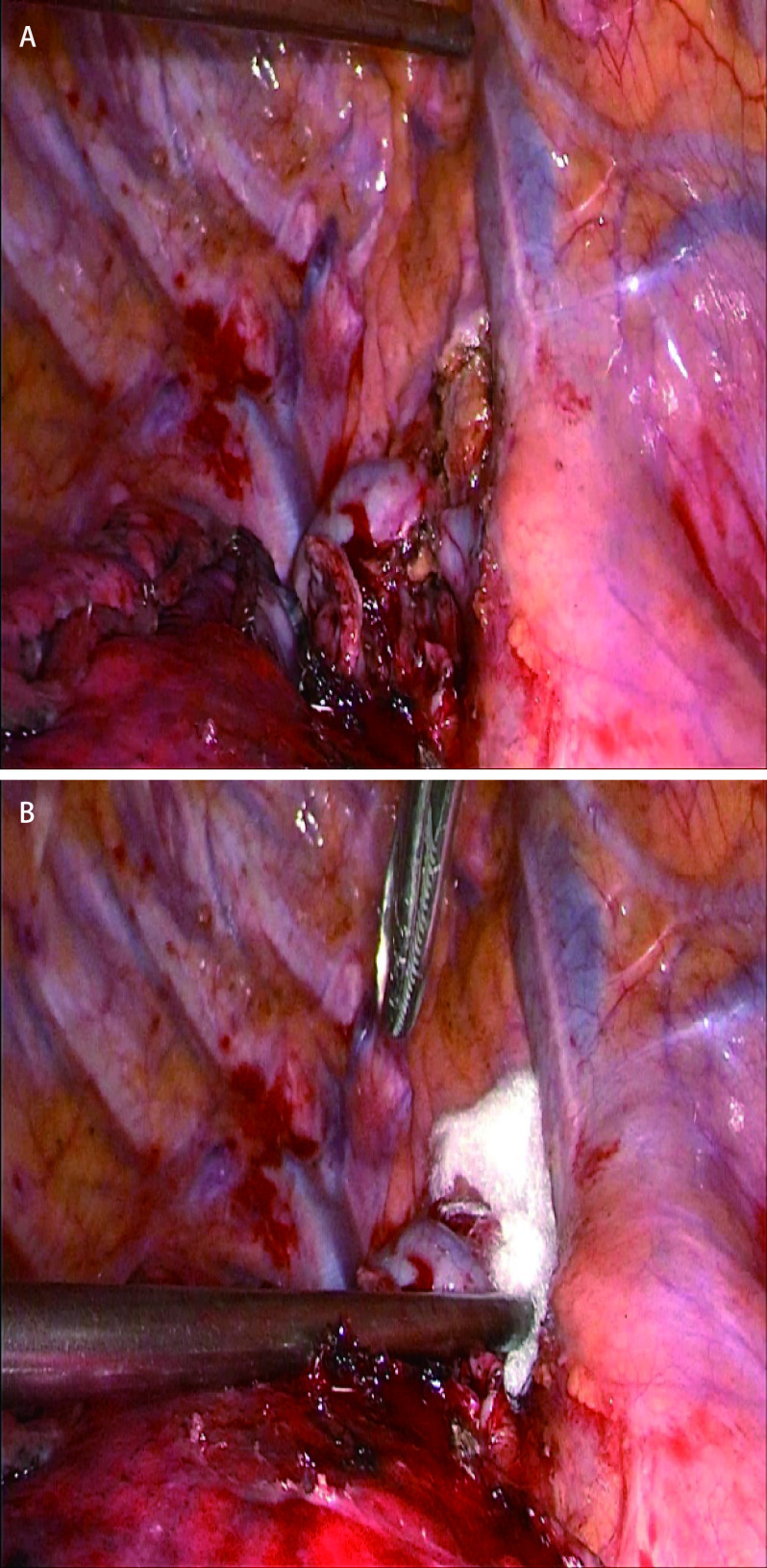
典型病例。A：淋巴结清扫；B：放置止血材料。 Typical case. A: lymph node dissection; B: place hemostatic material.

## 3 讨论

止血是手术安全的重要保证，胸外科加速康复外科的发展离不开术中彻底的止血，围术期的出血会导致许多并发症，如：低血容量性休克、器官功能不全等^[8]^，延长住院时间，增加住院期间费用。胸腔镜辅助下的肺癌根治术是现今胸外科发展最为成熟的术式，与此同时，可吸收止血材料的研发技术不断更新，以适应腔镜手术中的止血需要^[9-11]^。雪花速即纱与传统纱布类止血材料具有不同塑形工艺，由针冲压成片而非编织而成，厚韧紧实，易于操控，可在镜下灵活操控，易于重置调整和安放。本研究目的为评估可吸收再生氧化纤维素（雪花速即纱）在胸腔镜辅助肺癌切除术中的止血效果。

本研究发现在去除患者个体因素及解剖因素的情况下，在胸腔镜下行肺癌根治术中使用可吸收止血材料的患者并未出现明显的术后并发症。使用可吸收再生氧化纤维素的患者术后第一个24 h引流量、总引流量均达到了满意的临床效果，拔管时间也较短。因此，规范使用止血材料有利缩短患者住院时间，减少围术期引流量，有利于患者快速康复，早日出院。

结合本中心丰富的临床经验以及该回顾性研究，我们认为，可吸收再生氧化纤维素在减少肺癌手术围术期出血方面有较大优势，尤其是减少肺断面、淋巴结清扫处及粘连胸腔创面处出血。现今，胸外科微创手术成为主流，各类微创器械发展进入了成熟的平台期，腔镜下各项操作更为方便，止血材料也在飞速发展，从明胶海绵到止血胶水，再到雪花速即纱，都有利于减少围术期出血，降低出血并发症的风险^[12]^。一项随机对照试验评估了新型流体明胶（Floseal®）在肺切手术中的止血效果，结果显示，与传统电凝止血相比，流体明胶组术中止血速度明显更快。文献报道流体明胶组的24 h、48 h和72 h胸腔引流量分别为（392.8±125.9）mL、（299.1±121.4）mL和（221.4±109.2）mL^[13]^。本研究病例术后3日引流量分别为（212.4±105.3）mL、（210.1±116.9）mL和（143.4±96.1）mL，略低于文献报告值。

病例回顾中未见围术期严重并发症，但文献仍有报道可吸收止血材料相关过敏引起分布性休克的案例，对于可吸收止血材料的应用我们应该明确其使用规范，适当应用。在本中心开展的术式中，可吸收止血材料发挥着重大作用，尤其是手术难度较大的情况下，术前行新辅助治疗的患者中，可吸收止血材料是手术成功不可或缺的一环。在外科快速康复理念的大背景下，本中心致力于加速康复外科的推进，围术期出血的防范是快速康复的重大保障。

既往文献报道，可吸收再生氧化纤维素在肝脏切除术、食管癌根治术中的止血效果好，但无在腔镜肺部分切除术中应用的相关报道。本研究是观察性研究，因此仍需前瞻性、对照性研究进一步评估可吸收再生氧化纤维素的有效性。

## References

[1] ChenWQ, Zheng RS, Baade D (2015). Cancer statistics in China, 2015. CA Cancer J Clin.

[2] Siegel RL, Miller KD, Jemal A (2010). Cancer Statistics, 2017. CA Cancer J Clin.

[3] Ettinger DS, Aisner DL, Wood DE (2018). NCCN guidelines insights: non-small cell lung cancer, version 5.2018. J Natl Compr Canc Netw.

[4] Yan TD, Black D, Bannon PG (2009). Systematic review and meta-analysis of randomized and nonrandomized trials on safety and efficacy of video-assisted thoracic surgery lobectomy for early-stage non-small-cell lung cancer. J Clin Oncol.

[5] Cui F, Liu J, Li S (2016). Tubeless video-assisted thoracoscopic surgery (VATS) under non-intubated, intravenous anesthesia with spontaneous ventilation and no placement of chest tube postoperatively. J Thorac Dis.

[6] Brunelli A, Thomas C, Dinesh P (2017). Enhanced recovery pathway versus standard care in patients undergoing video-assisted thoracoscopic lobectomy. J Thorac Cardiovasc Surg.

[7] Azadani Ali N, Matthews Peter B, Ge L (2009). Mechanical properties of surgical glues used in aortic root replacement. Ann Thorac Surg.

[8] Chetter I, Stansby G, Sarralde J (2017). A prospective, randomized, multicenter clinical trial on the safety and efficacy of a ready-to-use fibrin sealant as an adjunct to hemostasis during vascular surgery. Ann Vasc Surg.

[9] Zhang Y, Song D, Huang H (2017). Minimally invasive hemostatic materials: tackling a dilemma of fluidity and adhesion by photopolymerization *in situ*. Sci Rep.

[10] Schenk Worthington G, Burks Sandra G, Gagne Paul J (2003). Fibrin sealant improves hemostasis in peripheral vascular surgery: a randomized prospective trial. Ann Surg.

[11] Li S, Zhou K, Che G (2017). Enhanced recovery programs in lung cancer surgery: systematic review and *meta-*analysis of randomized controlled trials. Cancer Manag Res.

[12] Miyamoto H, Sakao Y, Sakuraba M (2010). The effects of sheet-type absorbable topical collagen hemostat used to prevent pulmonary fistula after lung surgery. Ann Thorac Cardiovasc Surg.

[13] D'Andrilli A, Cavaliere I, Maurizi G (2015). Evaluation of the efficacy of a haemostatic matrix for control of intraoperative and postoperative bleeding in major lung surgery: a prospective randomized study. Eur J Cardiothorac Surg.

